# Editorial: Reproductive genomics

**DOI:** 10.3389/fgene.2022.1002458

**Published:** 2022-08-23

**Authors:** Rong Liu, Yan Yun, Wenjie Shu, Xi Wang, Mengcheng Luo

**Affiliations:** ^1^ Hubei Provincial Key Laboratory of Developmentally Originated Disease, Department of Human Histology and Embryology, Taikang Medical School (School of Basic Medical Sciences), Wuhan University, Wuhan, China; ^2^ Department of Microbiology and Molecular Genetics, University of California, Davis, CA, United States; ^3^ Beijing Institute of Microbiology and Epidemiology, Beijing, China; ^4^ State Key Laboratory of Reproductive Medicine, Nanjing Medical University, Nanjing, China

**Keywords:** reproductive biology, reproductive medicine, reproductive genomics, reproductive genetics, single-cell sequencing, omics, preimplantation genetic testing, risk factors

In all sexually reproducing animals, a new life starts with a zygote, which derives from the successful fusion of a mature oocyte with a mature sperm. Despite the complicated journey taken for a zygote to become a well-developed individual, the production of the mature gametes (i.e., sperms and oocytes) also relies on the normally programmed gametogenesis of both the male and female. This early development is comprised of many complicated and delicate processes, accompanying with numerous molecular regulation and metabolic changes. For example, PLZF (OMIM: 176797) and c-KIT (OMIM: 164920) are associated with self-renewal and differentiation of spermatogonial stem cells (Buaas et al., 2004; Costoya et al., 2004; Zhang et al., 2013), DNA-binding protein inhibitor ID2 (OMIM: 600386) is the key transcription factor in mouse primordial follicle formation (He et al., 2021a). Reprogramming of epigenome is essential for the early embryo development (Xia and Xie, 2020; Xu R. et al., 2021).

There is an urgent need to obtain a better understanding of the fundamental reproductive biology. This need is driven by two factors. First, there has been a decline in birth rate during the past half century (Skakkebaek et al., 2022). Second, infertility in human caused by genetic defects is a barrier for many couples (Zorrilla and Yatsenko, 2013; Krausz and Riera-Escamilla, 2018). Recently, considerable efforts have been devoted to achieving better understanding of the molecular networks, regulatory programs, and germline-soma or soma-soma communications during gametogenesis and embryogenesis. In particular, the rapid advancement of high-throughput sequencing technologies and ultra-low-input (or even single-cell) omics approaches have accelerated novel discoveries in reproductive development e.g., (Wang et al., 2018; Qiao et al., 2020; Xu K. et al., 2021; He et al., 2021b; Tyser et al., 2021; Yan et al., 2021; Wu et al., 2022; Xiong et al., 2022). Towards this direction, Qian et al. profiled the transcriptomes of 14,315 single testicular cells from adult zebrafish testes by single-cell RNA sequencing (scRNA-seq) using the 10x Genomics Chromium platform, identified ten distinguishable cell types with novelly revealed marker genes, and characterized interactions between somatic cells and germ cells through ligand-receptor analysis. This study provides an important resource for the studies of spermatogenesis in zebrafish, as well as mechanisms associated with human male infertility by using zebrafish as a model organism (Hoo et al., 2016). In addition to the application of single-cell omics to reproductive biology, Dodlapati et al. developed artificial intelligence (AI)-based novel computational approaches for the imputation of single-cell DNA methylome profiles with ultra-low coverage. The high effectiveness of the new algorithm was demonstrated by its application to bovine oocytes and early embryos, highlighting the potential for reconstructing epigenome-mediated transcriptional regulatory networks at the single-cell level in early animal development. Focusing on gene expression at the RNA level, RNA-sequencing was utilized to study oocyte development between different sex chromosome complement (by Yamazaki et al.) and between species (by Zhang et al.). More specifically, Yamazaki et al. compared transcriptomes among oocytes derived from XX, XO (i.e., monosomy X) and sex-reversed XY female mice (Vaz et al., 2020; Yamazaki et al., 2022) along follicular growth up to maximum size allowed without significant morphological differences. They found losing a copy of X chromosome is the dominant effect on gene expression changes in only XO oocytes, whereas the transcriptome landscape in XY oocytes is associated with the expression of Y-linked genes. Meanwhile, Zhang et al. performed comparative transcriptomic analysis and weighted gene co-expression network analysis (WGCNA) to compare the transcriptomes of donkey oocytes to that of cattle, sheep, pigs, and mice. They uncovered unique aspects of gene expression of donkey oocyte development from germinal vesicle (GV) to metaphase II (MII), thus providing new insights into the key regulators in donkey oocyte development.

Besides the efforts made in the fundamental research of reproductive biology, the accessibility of high-throughput sequencing and accumulated knowledge on inherited diseases have led to an extensive development and application of new technologies in screening, diagnosis and prevention of reproduction and pregnancy-related diseases (Hu et al., 2011; Hou et al., 2013). In order to develop an efficient and cost-effective method for screening pathogenic genes in infertility patients, Yuan et al. designed a target-sequencing panel containing 22 female infertility-related genes and applied such genetic screening to 68 patients with primary infertility or recurrent pregnancy loss. The authors demonstrated that the target-sequencing approach can be applied not only for genetic screening in IVF clinics, but also for uncovering novel pathogenic variants in the infertility-related genes. To reduce the risk of pregnancy loss, Pei et al. developed a clinically applicable method for detecting chromosomal reciprocal translocations in human embryos by long-read Nanopore sequencing and breakpoints region analysis. With this approach, the accurate and precise identification of balanced translocations provides a way of selecting embryos with normal karyotype for transfer into the uterus. Due to the association of reciprocal translocations and reproductive problems (Morin et al., 2017), aiming at providing more appropriate genetic counseling for couples with autosomal reciprocal translocations on their chances of producing normal blastocysts, Xie et al. evaluated several factors that may affect the unbalanced rearrangement of reciprocal translocations. The authors analyzed the meiotic segregation patterns in 10,846 blastocysts from 2,871 preimplantation genetic testing cycles of reciprocal translocation carriers, and found decreased proportions of alternate segregation in blastocysts when an Acr-ch, female sex, and lower TAR1 were involved. To avoid aneuploid embryo transfers in patients with implantation failure and pregnancy loss, Chen et al. analyzed the correction of time lapse-based blastocyst morphological scores (TLBMSs) with mosaic levels. In the study, high-resolution next-generation sequencing (NGS) was applied to evaluate the mosaic level of a blastocyst, and time-lapse embryo assessments were refined at a uniform time-point. With 918 biopsied blastocysts, Chen et al. showed that the redefined blastocyst morphology components and the converted TLBMSs are significantly correlated with all of the threshold levels of mosaicism. Expanded carrier screening (ECS) is applied to identify at-risk couples who carry heterozygous disease-causing variants and to avoid birth defects (Martin et al., 2015). Since the current expanded carrier sequencing panel cannot fully cover the variant spectrum in the East Asian population (Guo and Gregg, 2019), Tong et al. aimed to reveal the carrier spectrum in the Chinese population and to delineate an expanded carrier gene panel suitable for Chinese people. They screened 2,234 couples and found 94.5% of them were carriers of at least one disease-causing variant, and at-risk couple rate was 9.8%, highlighting the necessity of establishing a Chinese population-tailored ECS gene panel and conducting ECS for couples before receiving assisted reproductive technology. Serving as a successful example of preimplantation genetic testing (PGT), Ren et al. reported a case of preventing the transmission of a disease-causing *IL2RG* (OMIM: 308380) variant in a family. This variant will cause X-linked severe combined immune deficiency (SCID-X1), which is a recessive monogenic hereditary disease. In this case, Sanger sequencing for validating the mutated allele and linkage analysis based on single nucleotide polymorphism (SNP) haplotype via NGS were performed simultaneously. The authors then transferred an embryo without copy number variation and inherited variants at the *IL2RG* gene, and saw a healthy girl was born finally. This case demonstrates the usefulness of PGT in preventing mutated allele transmission.

Related to the assisted reproductive technology, Wang et al. compared different methods for cryopreserving human ejaculated and testicular spermatozoa by analyzing molecular and metabolic features related to sperm quality, and demonstrated that direct −80 °C freezing could be a viable alternative to liquid nitrogen vapor freezing for short-term human sperm storage. In recent years, totipotent or pluripotent stem cells have been gradually used to model embryonic development for mechanism studies. Towards a better understand of the maintenance and developmental potential of human extended pluripotent stem (hEPS) cells, An et al. constructed inducible WDR36 (OMIM: 609669) knockdown and WDR36-overexpressing hEPS cell lines, and their data demonstrated that WDR36 safeguards the self-renewal and pluripotency of hEPS cells.

So far, accumulating studies, including those in this Research Topic, have gained many novel insights into spatiotemporal regulatory networks during gametogenesis and embryonic development, leading to applications such as molecular diagnosis of various reproductive diseases. To better diagnose and treat infertile couples and to partially address the declining birth rate, both fundamental and translational research is still desired. We anticipate that continued development and refinement of technologies will continue to drive research in this field.
